# Changes of circulating Th22 cells in children with hand, foot, and mouth disease caused by enterovirus 71 infection

**DOI:** 10.18632/oncotarget.14083

**Published:** 2016-12-21

**Authors:** Dawei Cui, Fengyun Zhong, Jie Lin, Yidong Wu, Quan Long, Xianzhi Yang, Qiaoyun Zhu, Li Huang, Qifen Mao, Zhaoxia Huo, Zhe Zhou, Guoliang Xie, Shufa Zheng, Fei Yu, Yu Chen

**Affiliations:** ^1^ Department of Laboratory Medicine, First Affiliated Hospital, College of Medicine, Zhejiang University, Hangzhou, China; ^2^ Key Laboratory of Clinical *In Vitro* Diagnostic Techniques of Zhejiang Province, Hangzhou, China; ^3^ Department of General Surgery, the Second Affiliated Hospital of Soochow University, Suzhou, China; ^4^ Department of Clinical Laboratory, Center of Community Health Service of Qingbo Street, Hangzhou, China; ^5^ Clinical Laboratory, Hangzhou Children’s Hospital, Hangzhou, China

**Keywords:** HFMD, EV71, circulating Th22 cell, circulating Th17 cell

## Abstract

Interleukin (IL)-22^+^CD4^+^T (Th22) cells play crucial roles in the pathogenesis of autoimmune diseases and infectious diseases, although the role of Th22 cells remains largely unclear in children with hand, foot, and mouth disease (HFMD) caused by enterovirus 71 (EV71). This study aims to explore the role of circulating IL-22^+^IL-17A^−^CD4^+^T (cTh22) cells in children with EV71-associated HFMD. We found that during the acute stage of illness, the frequencies of cTh22 and circulating IL-22^+^IL-17A^+^CD4^+^T (IL-22^+^cTh17) cells in CD4^+^T cells infrom affected patients, and especially in severely affected patients, were significantly higher than in healthy controls (HC). The major source of IL-22 production was cTh22 cells, partially from cTh17 cells. Moreover, the protein and mRNA levels of IL-22, IL-17A, IL-23, IL-6, and TNF-α were significantly different among the mild patients, severe patients and HC, as well as AHR and RORγt mRNA levels. A positive correlation was found between plasma IL-22 levels and cTh22 cell frequencies, and cTh17 cell and IL-22^+^ cTh17 cell frequencies. Furthermore, the frequencies of cTh22 were significantly decreased in the convalescent patients. Our findings indicated that cTh22 cells could play critical roles in the pathogenesis of EV71 infection, and are potential therapeutic targets for patients with EV71-associated HFMD.

## INTRODUCTION

Hand, foot and mouth disease (HFMD) is a common infectious disease in young children, and outbreaks occur annually worldwide [1–2]. There are over one million HFMD cases in the world and 200 cases of mortality since 2008, and approximately 9 million cases of HFMD have been reported between 2008 and 2013 in China [3–5]. Enterovirus 71 (EV71) is one of the most common etiologies of HFMD, and EV71-associated HFMD has been increasingly reported in Asia-Pacific regions such as Malaysia, Japan, Singapore, China, and South Korea among young children less than five years of age since 1997 [6–10]. EV71 infection is a serious and growing public health issue in the world. In most instances, HFMD is mild and self-limited. However, EV71 infection is considered as the major cause of clinically severe HFMD cases with severe pulmonary edema, neurological complications, cardiopulmonary failure, and death [6, 11–13]. Furthermore, the pathogenesis of severe HFMD caused by EV71 infection remains unknown. The disorders of cellular and humoral immune responses may play important roles in the pathogenesis of EV71-related severe diseases.

Previous reports have shown that abnormal changes in Th1, Th2, Th17, T follicular helper (TFH), and T regulatory (Treg) cells and associated cytokines such as interleukin (IL)-6, IL-17A, IL-21, and tumor necrosis factor alpha (TNF-α) are all involved in the pathogenesis of EV71-associated HFMD [4, 13–16]. Recently, interleukin (IL)-22-producing CD4^+^T (Th22) cells have been clearly described as a new subset of CD4^+^T cells characterized by production of IL-22 without IL-17. T, and these cells play crucial roles in the pathogenesis of multiple diseases such as cancers, autoimmune diseases and infectious diseases [17–20]. IL-22, a member of the IL-10 family or IL-10 superfamily, plays an important role in various inflammatory diseases and infectious diseases. IL-22 is primarily produced by obvious immune cells, such as Th22, Th17, NKT, and macrophages. Th22 cells are a major source of IL-22 in many diseases [21–23]. Moreover, the proliferation and differentiation of Th22 cells are derived by activated transcription factors such as aryl hydrocarbon receptor (AHR) and retinoid-related orphan nuclear receptor-γt (RORγt) that are required for some cytokines including IL-6, TNF-α, IL-23 and IL-1β [22–25]. However, the role of circulating Th22 (cTh22) cells is largely unknown in children with EV71-associated HFMD.

In this study, we reported that the frequencies of circulating IL-22^+^IL-17A^−^CD4^+^T (cTh22) cells, circulating IL-17A^+^CD4^+^T (cTh17) cells and IL-22^+^IL-17A^+^CD4^+^T cells (IL-22^+^cTh17) in CD4+T cells from peripheral blood mononuclear cells (PBMCs) during the acute stage of children with EV71-associated HFMD were significantly increased compared to healthy controls (HC); however, the percentages of cTh22, IL-22^+^cTh17 and cTh17 cells in the mild cases of HFMD were notably decreased compared to the severe cases. The major source of intracellular IL-22 secretion was from cTh22 cells, followed by cTh17 cells in PBMCs from both the patients and HC. Interestingly, the frequencies of cTh22 in total intracellular IL-22^+^ cells obtained from the cases with EV71 infection were obviously lower; conversely, the percentages of cTh17 cells in total intracellular IL-22^+^ cells were higher in patients who were infected than in HC. The frequencies of cTh22 or cTh17 cells in total intracellular IL-22^+^cells were significantly different between the mild and severe patients. Furthermore, most of the proteins and related mRNA levels of IL-22, IL-17A, IL-23, IL-6, and TNF-α were elevated significantly in the cases with EV71-associated HFMD compared to HC. We also found significantly elevated AhR and RORγt mRNA levels in EV71-infected HFMD patients. Some differences were observed between mild and severe cases. Additionally, plasma levels of IL-22 were positively correlated with percentages of cTh22 cells; similarly plasma IL-17A levels were positively correlated with cTh17 cells. The frequencies of cTh22 and cTh17 cells were significantly decreased in the convalescent cases. Taken together, these findings suggest that cTh22 cells and associated cells and effector molecules are possibly involved in the immunopathogenesis of EV71 infection, which offers potential therapeutic targets for children with EV71-associated HFMD.

## RESULTS

### Clinical characteristics of EV71-associated HFMD patients

EV71, a positive-stranded RNA virus, is considered a common etiology of HFMD (a self-limited disease) among children less than five years of age and is normally diagnosed using a PCR assay [1, 27]. Most of the HFMD cases are mild, while severe cases are predominantly attributed to EV71 infection [12–13]. In this study, a total of 56 EV71-associated HFMD patients were confirmed using the enterovirus 71 nucleic acid detection kit for detection in at least one of the clinical samples recruited from May 2014 to October 2015. According to the definition for mild and severe HFMD, 32 patients (18 males and 14 females) were classified as mild, and 24 patients (13 males and 11 females) were classified as severe. The youngest patient was 8 months old, the oldest patient was 72 months old, and the majority of the patients were less than 5 years of age. Most of the patients had fever (mild: 31 [96.9%], severe: 24 [100%]). All patients had rashes on the hands and feet and had vesicles in the mouth. The average age of the mild patients (30.3±16.8 months) was older than the average age of the severe patients (23.3±9.8 months) (*P* = 0.2438). The number of hospitalized days for the mild cases (5.0±1.4 days) was significantly less than the severe patients (16.7±4.1 days) (*P* < 0.0001). The number of peripheral WBCs and serum levels of CRP, CK-MB, AST, and LDH were notably elevated in the mild and severe patients compared to HC. The number of peripheral WBCs and plasma levels of LDH and CK-MB in the severe patients were notably higher than in the mild patients during the acute phase of HFMD (*P* < 0.05). However, Ct value was not significantly different between mild (range of Ct value: 21.42~32.57) and severe (range of Ct value: 20.65~31.86) patients (*P* = 0.275) (Table 1).

**Table 1:** Clinical characteristics of 56 recruited cases with EV71-associated HFMD

Group	Age (Months)	Gender (M/F)	Hospitalized days	Fever (>37.5◦C)	Rash	Laboratory results					
						WBC (×109/L)	CRP (mg/L)	CK-MB (U/L)	AST (mmol/L)	LDH (U/L)	Viral Titer (Ct value)
HC	39.5±18.7	26/20	no	no	no	6.42±0.91	0.15±0.10	19.95±3.73	26.75±6.25	275.19±56.85	no
Mild	30.3±16.8	18/14	5.0±1.4	31 (96.9%)	32(100%)	9.74±3.28	6.87±3.37	41.94±11.96	36.87±8.69	326.31±68.15	25.65±4.62
Severe	23.3±9.8	13/11	16.7±4.1	24 (100%)	24 (100%)	13.62±5.38	8.79±4.66	40.58±9.84	37.58±11.22	374.87±94.71	25.87±4.36

Note: data correspond to the arithmetic mean±SD. M/F: male/female; WBC: White blood cell; CRP: C-reactive protein; CK-MB: Creatine kinase-MB; AST: Aspartate aminotransferase; ALT: Alanine aminotransferase; LDH: Lactate dehydrogenase; HC: healthy controls; Ct: Cycle threshold. The Ct value defines as the number of cycles required for the fluorescent signal to cross the detection threshold, the low Ct value represents high viral titer, and high Ct value represents low viral titer. 56 Laboratory-confirmed HFMD children caused by EV71 infection were enrolled and classified into two groups depending on the clinical severity of disease and degrees of neurological damage.

### Increased frequency of cTh22 cells among patients with EV71-associated HFMD

**Figure 1:**
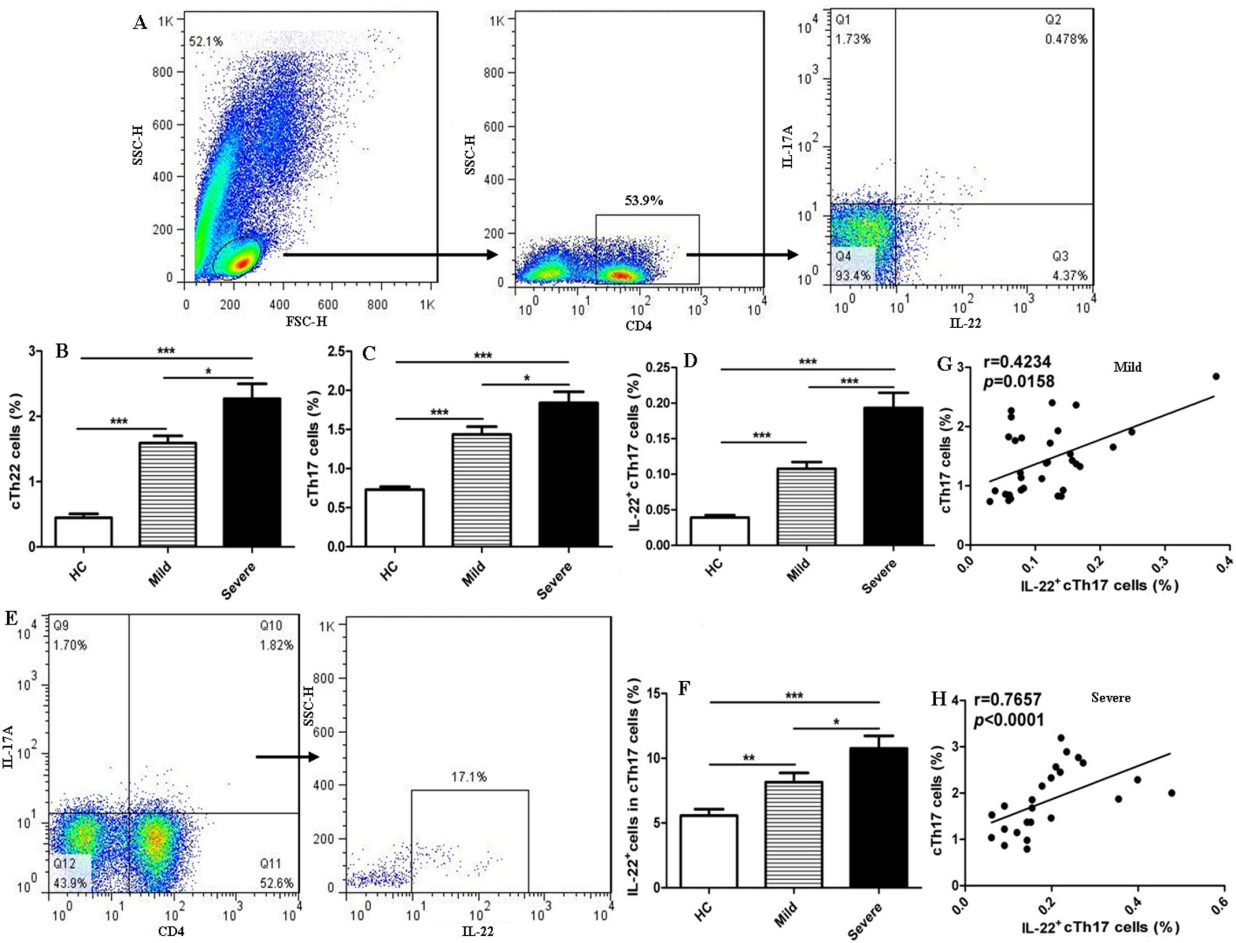
Increased frequencies of cTh22 and cTh17 cells in human CD4 **^+^**T cells of peripheral blood from patients with EV71-associated HFMD Human peripheral blood mononuclear cells (PBMCs) from 32 mild and 24 severe patients with EV71-associated HFMD and 46 healthy controls (HC) were isolated and stained with labeled antibodies and analyzed by flow cytometry as described in the Methods section. **A**. The cells were gated initially on lymphocytes and then on CD4^+^T cells, circulating IL-22^+^IL-17A^−^CD4^+^Th22 (cTh22) cells, circulating IL-17A^+^CD4^+^Th17 (cTh17) cells, and IL-22^+^IL-17A^+^CD4^+^T (IL-22^+^cTh17)cells in the total number of CD4^+^T cells. **B**.-**D**. The analysis of the percentages of cTh22 cells, cTh17 cells and IL-22^+^cTh17 cells in CD4^+^T cells from HC and mild and severe patients. **E**.-**F**. The proportion of circulating IL-22^+^ cells in cTh17 cells. **G**.-**H**. The correlation of cTh17 cells and IL-22^+^cTh17 cells in mild and severe cases. *, *p* < 0.05; **, *p* < 0.01; ***, *p* < 0.001; ns, no significant difference.

sMany evidences indicating that Th22 cells play a critical and complicated role in chronic inflammation and autoimmune diseases such as HIV, psoriasis, rheumatoid arthritis (RA), systemic lupus erythematosus (SLE), and diabetes [31–34]. Additionally, elevated IL-22-producing Th17 cells are associated with disease activity in various inflammatory diseases, such as psoriasis, asthma, and multiple sclerosis (MS) [35–37]. However, the role of Th22 cells has not yet been elucidated in patients with EV71-associated HFMD. To explore the potential role of cTh22 cells in patients with EV71-associated HFMD, the frequency of cTh22 cells was analyzed using a flow cytometry assay (Figure 1). CD4^+^T cells in human PBMCs were gated, and intracellular IL-22^+^IL-17A^−^T cells were analyzed to delimit cTh22 cells in peripheral blood from the mild or severe patients and HC (Figure 1A). The frequencies of cTh22 cells were significantly higher in severe patients compared to mild patients and HC, and the proportions of cTh22 cells were notably increased in mild patients compared to HC (Figure 1B). The frequencies of IL-22^+^cTh17cells or cTh17 cells were obviously increased in the mild and severe patients compared to HC and significantly different between the mild and severe patients (Figure 1C-1D). Interestingly, trace amounts of intracellular IL-22 from cTh17 cells were found in the HC and mild and severe patients (Figure 1E-1F). In addition, further analysis revealed a positive and modest correlation between cTh17 cells and IL-22^+^cTh17 frequenciesand but not IL-22^+^cTh17 cells and cTh22 cells in the mild and severe patients, respectively. No significant relationship in the frequencies of cTh22 and cTh17 cells was observed in this study.

### Circulating Th22 cells are a principal source of intracellular IL-22 in peripheral blood

**Figure 2:**
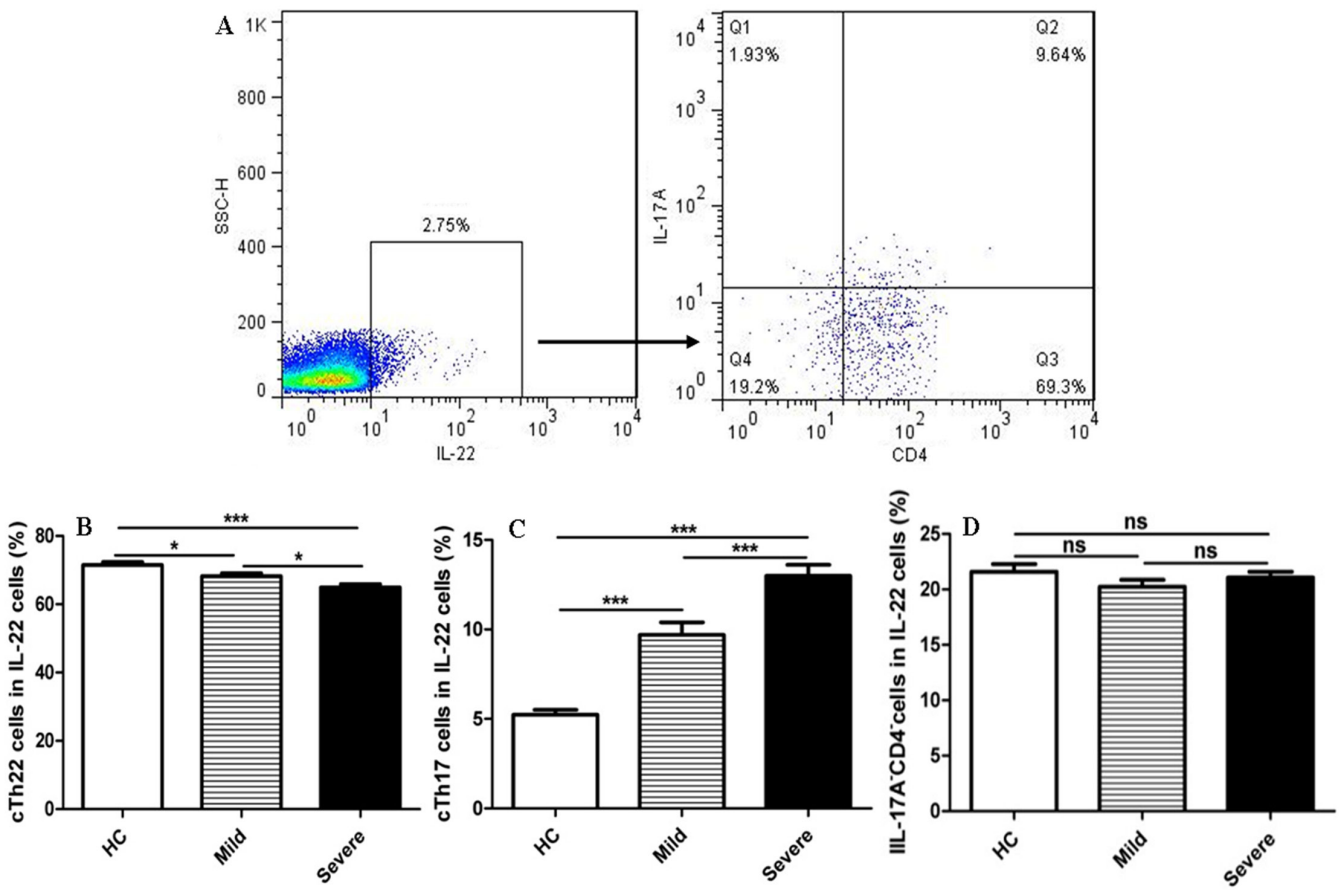
Circulating Th22 cells, a principal source of intracellular IL-22 in peripheral blood **A**. The cells were gated initially on IL-22^+^ cells and then on CD4^+^T cells and IL-17A cells. **B**. Circulating IL-22^+^IL-17A^−^CD4^+^Th22 (cTh22) cells. **C**. Circulating IL-22^+^cTh17 cells. **D**. IL-17A^−^CD4^−^cells in the total IL-22^+^ cells were analyzed in HC and mild and severe patients. *, *p* < 0.05; **, *p* < 0.01; ***, *p* < 0.001; ns, no significant difference

IL-22 is expressed by many immune cells, including innate and adaptive immune cells such as Th22, Th17, CD8^+^T, dendritic cells (DCs), and γδT cells, which principally originated from Th22 cells [19, 21, 25]. To determine whether the principal source of intracellular IL-22 was from cTh22 in peripheral blood obtained from the patients, the frequencies of cTh22, cTh17, and CD4^−^ cells were detected in gated intracellular IL-22 cells using flow cytometry analysis in this study (Figure 2). The highest proportion of total intracellular IL-22^+^ cells was expressed by cTh22 cells in the patients and HC, followed by IL17^−^CD4^−^cells and cTh17 cells (Figure 2A-2D).

Moreover, the frequencies of cTh22 cells in the total number of intracellular IL-22^+^ cells from severe patients were slightly lower than the frequencies in mild patients and HC, and the proportions of cTh22 cells in the intracellular IL-22^+^cells were also notably reduced in mild patients compared to HC. Conversely, the frequencies of cTh17 cells in the total number of intracellular IL-22^+^ cells from severe patients were significantly higher than the frequencies in mild patients and HC, and the proportions of cTh17 cells were also notably increased in mild patients compared to HC.

### High levels of plasma cytokines with increased cTh22 cells in EV71-associated HFMD cases

**Figure 3:**
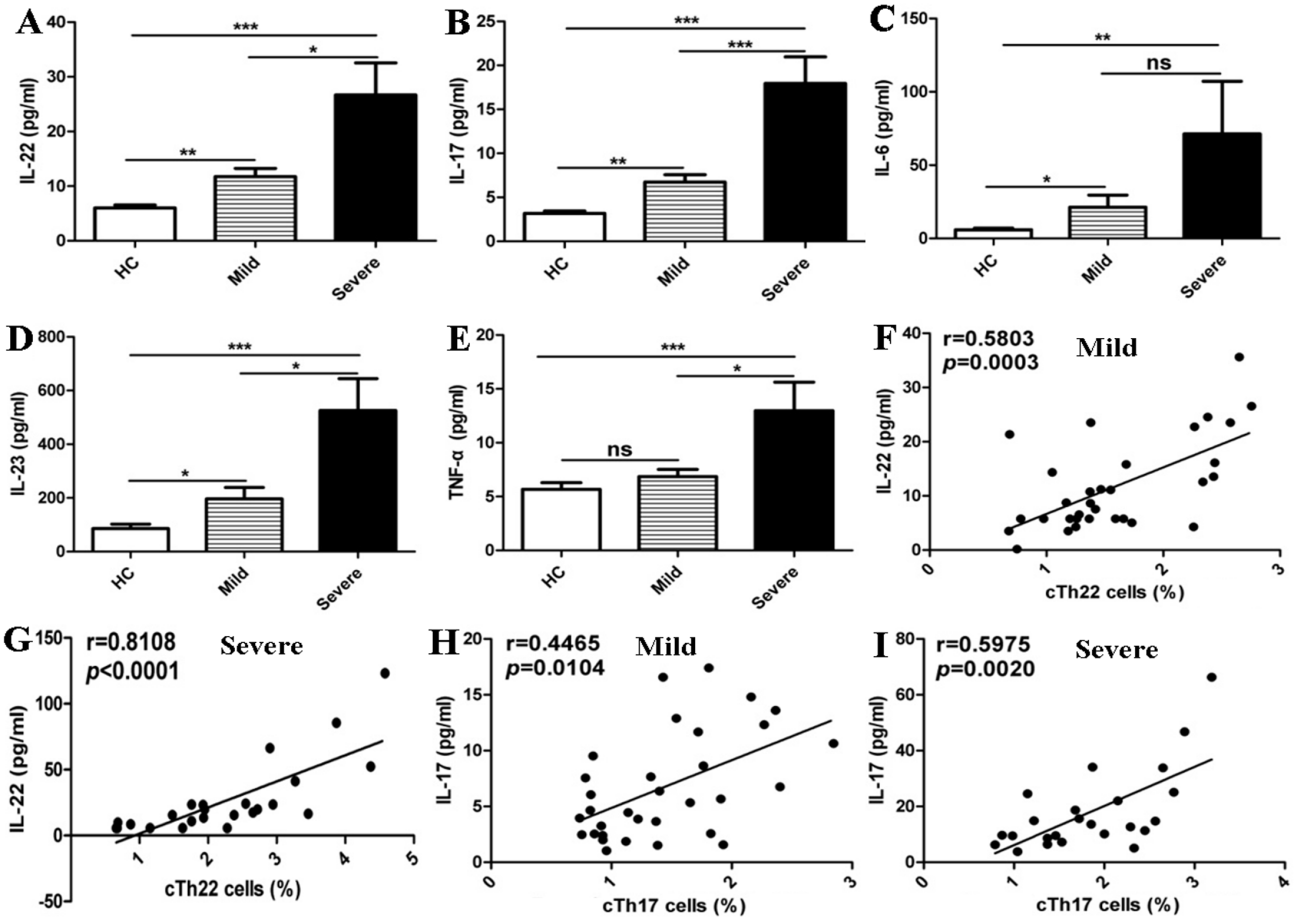
Elevated levels of cTh22-associated plasma cytokines in the patients with EV71-associated HFMD **A**.-**E**. Levels of plasma IL-22, IL-17, IL-6, IL-23 and TNF-α in HC and mild and severe patients. **F**.-**G**. Relationship of plasma IL-22 levels and the percentage of cTh22 cells in mild and severe patients, respectively. **H**.-**I**. Relationship of plasma IL-17 levels and the percentage of cTh17 cells in mild and severe patients, respectively. *, *p* < 0.05; **, *p* < 0.01; ***, *p* < 0.001; ns, no significant difference

IL-22 is principally released from Th22 cells and plays a critical role in inflammatory responses that occur in conditions such as autoimmune diseases, infections, and tumors [21, 25]. Naïve T cells can be differentiated into Th22 cells in the presence of TNF-α and IL-6 [17, 21, 38]. Moreover, IL-23 can promote the secretion of IL-22 by ILCs and Th17 cells [25, 39]. In this study, the plasma IL-22, IL-6, IL-17A and IL-23 levels were significantly higher in the patients than in HC, and no significant changes in plasma levels of TNF-α were observed between the mild patients and HC. Except for IL-6, all cytokine levels in the severe cases were notably higher than levels observed in the mild cases (Figure 3A-3E). Additionally, the plasma levels of IL-22 were positively correlated with the frequencies of cTh22 cells in the mild and severe patients (Figure 3F-3G). Furthermore, a modest and positive correlation was found between plasma IL-17A levels and the frequencies of cTh17 cells in the mild and severe patients (Figure 3H-3I).

### Increased mRNA expression of cTh22 cells is associated with cytokines and transcription factors in patients with EV71-associated HFMD

**Figure 4:**
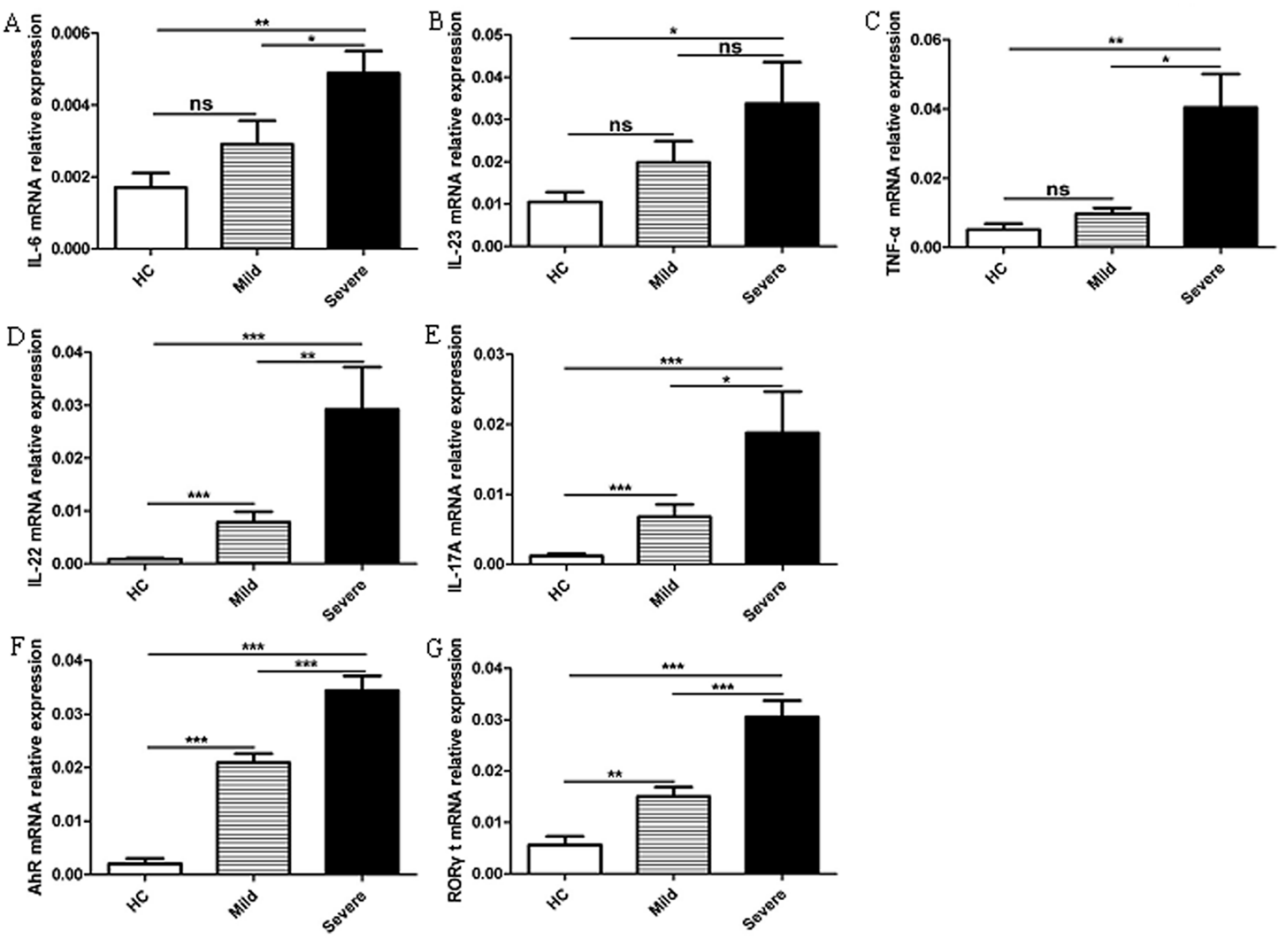
Increased mRNA expression of cTh22 cells is associated with cytokines and transcription factors in patients with EV71-associated HFMD **A**.-**C**. Human peripheral blood mononuclear cells (PBMCs) from 18 mild patients and 11 severe patients with EV71-associated HFMD and 11 healthy controls (HC) were isolated and analyzed using the PCR assay for IL-6, IL-23 and TNF-α mRNA expression. **D**.-**G**. The mRNA levels of IL-22, IL-17A, AHR and RORγt in CD4^+^T cells were analyzed using the PCR assay as described in the Methods section. *, *p* < 0.05; **, *p* < 0.01; ***, *p* < 0.001; ns, no significant difference

The cytokines TNF-α, IL-23 and IL-6 along with the transcription factors AHR and RORγt play crucial roles in the generation and differentiation of Th22 and Th17 cells [21, 25, 38, 40]. Th22 cells have biological functions that are dependent on IL-22 production, and Th17 cells require IL-17A [21–25]. In this study, we assessed the mRNA expression of cytokines (IL-23, IL-6, and TNF-α in PBMCs and IL-22 and IL-17A in CD4^+^T cells) and transcription factors (AHR and RORγt) in CD4^+^T cells from the peripheral blood of the mild patients (*n* = 18), severe patients (*n* = 11), and HC (*n* = 11). The IL-6, IL-23, and TNF-α mRNA expression levels were all significantly increased in the severe patients compared to HC, and a slight difference in IL-6 and TNF-α mRNA was observed between the mild and severe cases. However, no significant difference in IL-23 was observed between the mild and severe patients (Figure 4A-4C). The expression levels of IL-22, IL-17A, RORγt, and AHR mRNA were all significantly higher in the mild and severe patients compared to HC, with a notable difference observed between the mild and severe patients (Figure 4D-4G).

### Alterations in the frequencies of cTh22 cells and cTh17 cells in the convalescent patients with EV71-associated HFMD

**Figure 5:**
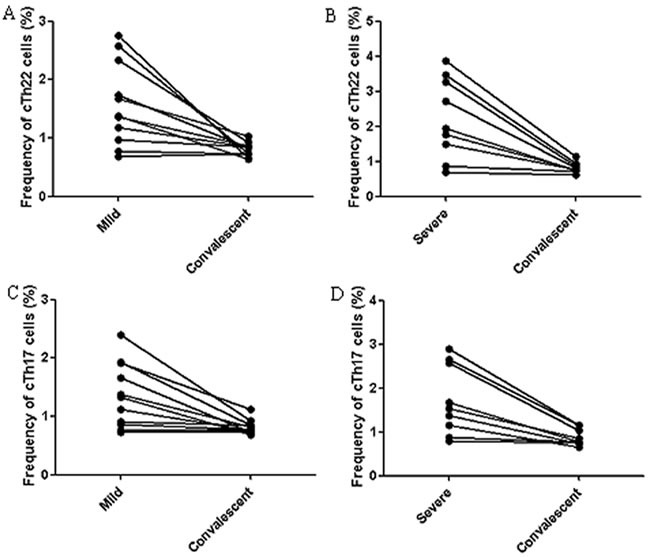
Alterations of cTh22 cells and cTh17 cells in the acute and convalescent patients with EV71-associated HFMD **A**.-**B**. The percentage changes in cTh22 cells in PBMCs from mild **A**. and severe **B**. patients. **C**.-**D**. The percentage changes in cTh17 cells in PBMCs from mild **C**. and severe **D**. patients

The frequency of cTh22 and cTh17 cells in PBMCs of the convalescent patients with mild HFMD (*n* = 11) and severe HFMD (*n* = 9) were analyzed using flow cytometry. We observed that the frequencies of cTh22 cells and cTh17 cells in PBMCs of convalescent patients were markedly decreased compared to the acute mild (*n* = 8) and severe (*n* = 7) patients, respectively. However, three mild and two severe patients showed no significant difference in the percentages of cTh22 cells and cTh17 cells between the acute and convalescent patients (Figure 5A-5D).

## DISCUSSION

Hand, foot, and mouth disease (HFMD) is a common childhood illness caused by serotypes of the enterovirus A species of the Picornaviridae family [1–3]. HFMD predominantly affects young children less than 5 years of age, characteristically causes acute illness with a duration of approximately one week, and most patients show self-limiting illness with typical symptoms including fever, skin eruptions on the hands and/or feet, and vesicles in the mouth [5–8]. However, a small number of patients with HFMD may develop neurological and systemic complications that can be fatal, particularly in patients infected by EV71 [11–12]. In the current study, all patients had positive EV71 virus confirmed using PCR assay for clinical samples. Typically, clinical features of the patients such as age, fever, and rashes were associated with the clinical diagnosis of EV71-associated HFMD, and number of hospitalized days was significantly different between the mild and severe patients, which was consistent with previous reports [2–3]. Moreover, laboratory evidence showed that WBC count and plasma levels of CRP, CK-MB, AST, and LDH were different between the mild and severe patients during the acute phase of EV71-associated HFMD. These findings implied that inappropriate inflammation may be involved in the progression of EV71-associated HFMD, which was consistent with previous reports [3, 6, 41]. The Ct value representing EV71 viral titer was not significantly changed between the mild and severe patients, which indicated that viral titer was not obviously associated with mild and severe characteristics. We speculated that the clinical characteristics of HFMD might be closely related with perineural invasion of EV71, which was consistent with previous reports [4–7].

Previous studies have indicated that both cellular and humoral immune responses, such as B cells and T cells including Th17, Th1, Th2, and CD8^+^T cells, EV71-antibody, and associated cytokines play important roles in animal models with EV71 infection [4, 15, 16]. Moreover, EV71 infection induced an inflammatory response and even a systemic dysregulation of the immune system in humans [41–42]. Recent studies show that Th22 cells, identified as a distinct subset of the Th cell family by specific production of IL-22, play crucial roles in regulating immune responses and are closely associated with multiple diseases, such as skin inflammation, infections, autoimmune diseases, and tumors [21–25]. Increased frequencies of circulating Th22 and Th17 cells have been identified in patients with psoriasis and contribute to cutaneous inflammation and systemic inflammatory disease that occurs in patients with psoriasis, and these were decreased after treatment of patients with psoriasis [35, 38]. Circulating Th22 and Th17 cells were significantly increased after successful antiretroviral therapy (ART) treatment for patients with HIV infection, which indicates that expansion of cTh22 and cTh17 can provide an immunological advantage for inhibiting HIV-1 infection [31]. Additionally, an increased frequency of cTh22 and cTh17 cells in RA patients is positively correlated with rheumatoid factor and disease severity [19, 32]. These reports demonstrate that Th22 and Th17 cells play important roles in various diseases, which possibly may aggravate or attenuate disease severity. However, the potential role of cTh22 cells and related cytokines in the patients with EV71-associated HFMD is not yet known.

In the present work, we first examined the frequency of cTh22 cells in mild and severe patients with EV71-associated HFMD. The results indicated that the frequency of cTh22 and cTh17 cells in CD4^+^T cells from mild and severe patients during the acute stage of EV71 infection was significantly higher than the frequency of these cells in HC, and severe patients differed significantly from mild patients in the percentage of cTh22 and cTh17 cells, respectively. However, the percentages of cTh22 and cTh17 cells were decreased in major convalescent patients. Expansion of cTh17 cells was consistent with previous reports [15–16]. These findings suggested that expansion of cTh22 and cTh17 cells was possibly driven by EV71 infection. Interestingly, a small proportion of circulating IL-22^+^cTh17 cells was observed in the patients and HC, and a significant discrepancy was found among the mild and severe patients and HC in this study. Moreover, a positive correlation was found between IL-22^+^cTh17and cTh17 cells in the mild and severe patients, respectively, which implies that cTh17 can secrete little IL-22, which is consistent with previous reports [23, 24, 36]. The Ct value of EV71 in mild or severe patients was not significantly associated with cTh22, cTh17 and IL-22^+^cTh17 cells (data not shown), respectively. These findings clearly indicated that expansion of cTh22 and cTh17 cells played critical roles in the EV71-induced immune responses and was involved in the disease severity, but impossibly associated with EV71 viral titer.

Accumulating evidence demonstrates that Th22 cells are characterized by IL-22 secretion, although IL-22 is also produced by other immune cells, such as Th17, CD8^+^T, NKT, γδT and macrophages [21, 25]. Whether the major source of intracellular IL-22 is from cTh22 cells in peripheral blood from the patients with EV71-associated HFMD is not yet clear. In our study, our results exhibited that intracellular IL-22 was mainly produced by cTh22 cells in the patients and HC. In addition, the percentages of cTh22 and cTh17 cells in intracellular IL-22 were significantly different in the mild patients, severe patients and HC. These findings indicated that the changes in the principal source of intracellular IL-22 were closely associated with EV71 infection and involved with the disease severity of EV71-associated HFMD. Moreover, the sources of intracellular IL-22 were affected by other immune cells (such as CD8^+^T, NKT, γδT and macrophages) in EV71-associated HFMD patients, which will be further explored in a future studyinvolving

IL-22 is an important cytokine in the regulation of tissue and cell responses during inflammation, and it plays a critical role in tissue or cell damage and repair of various autoimmune diseases, chronic inflammatory diseases, infectious diseases and cancers [21–26]. In the current study, significant differences in plasma levels of IL-22 were observed among HC and, mild and severe patients, which suggested that elevated levels of IL-22 cytokines might play an important role in the pathogenesis of EV71-associated HFMD, which was consistent with previous reports [29]. Subsequent correlation analysis of plasma levels of IL-22 and the frequencies of cTh22 cells further indicated that increased IL-22 levels might be predominantly secreted by cTh22 cells in the patients with EV71-associated HFMD. Inflammatory cytokines of TNF-α and IL-6 cooperate to induce the differentiation of Th22 cells from naïve CD4^+^T cells by a transcription factor (AHR). IL-23 can induce the production of IL-22 by ILCs, Th17, and γδT cells that are involved with transcription factors (AHR and RORγt) [28–30]. IL-6 and IL-23 are closely associated with generation and differentiation of Th17 cells by regulating the activation of RORγt. Th17 cells and IL-17A play important roles in autoimmune diseases, chronic inflammatory diseases, infectious diseases and cancers [16, 32, 35, 38]. In this study, correlation analysis showed an increase in the plasma levels of IL-17A and the frequencies of cTh17 cells, which also implied that the increased IL-17A expressions might be predominantly produced by cTh17 cells and associated with the pathogenesis of EV71-associated HFMD. Moreover, plasma levels of TNF-α, IL-6 and IL-23 cytokines were significantly higher in the EV71-associated HFMD patients than in HC, particularly in the severe patients, which was consistent with previous reports [4, 29, 30]. These findings indicated that increased plasma levels of cytokines (TNF-α, IL-6 and IL-23) possibly could explain the increased percentages of cTh22 and cTh17 cells and plasma levels of IL-22 and IL-17A in patients with EV71-associated HFMD, particularly in the severe patients. Additionally, PCR analyses further implied that the increased mRNA expression levels of cytokines (IL-22, IL-17A, TNF-α, IL-6 and IL-23) and transcription factors (AHR and RORγt) possibly contributed to expanding the number of cTh22 and cTh17 cells in patients with EV71-associated HFMD, particularly in the severe patients. However, we did not observe any positive or negative correlation between cytokine levels and frequency of cTh22 or cTh17 cells in HC in this study (data not shown), which implied that the balance of the internal environment based on the balance of various cells (such as Th22, Th17, NK, B cells) and cytokines (as IL-22, IL-17A, TNF-α, IL-6 and IL-23) *in vivo* contributes to the health of the body [24, 25, 28].

Once the harmonious balance of immune cells and cytokines of the hosts *in vivo* is broken by external pathogens such as viruses and bacteria, infectious diseases such as EV71-associated HFMD may possibly occur. In this study, the frequency of cTh22 and cTh17 cells was interestingly decreased in most of the patients with EV71-associated HFMD when they were in the convalescent stage after treatment with agents including the antiviral drug Ribavirin and other adjuvant therapies. Ribavirin can effectively inhibit the replication of the EV71 virus as described previously [43–44]. These results suggested that the antivirus drug Ribavirin and other adjuvant therapies contributed to clearing the EV71 virus and restored the balance of immune homeostasis *in vivo*.

In conclusion, our results showed that the frequencies of cTh22 cells and associated cells (cTh17), the expression of cTh22 cells and associated cytokines (IL-22, IL-17A, TNF-α, IL-6 and IL-23) and transcription factors (AHR and RORγt) were significantly increased in the patients with EV71-associated HFMD, particularly in severe patients compared to HC. This result suggests that the EV71 virus contributed to the production of multiple cytokines that induce generation and differentiation of Th22 and Th17 cells that further secrete IL-22 and IL-17A. Antiviral drug treatment might contribute to effectively reducing the frequencies of cTh22 and cTh17 cells that attenuate disease severity in patients with EV71-associated HFMD. Therefore, Th22 cells may play a critical role in the pathogenesis of EV71-associated HFMD, and could be considered as new therapeutic targets for EV71-associated HFMD patients in the future. The roles of Th22 cells in regulating inflammatory responses in the pathogenesis of EV71-associated HFMD will be further investigated *via* studies incorporating a large number of patients and animal models with EV71 infection.

## MATERIALS AND METHODS

### Case demographics

According to clinical criteria for the diagnosis and treatment of HFMD cases published by the Chinese Ministry of Health in 2009 [4, 26], a total of 56 inpatients less than 8 years of age with EV71-associated HFMD were enrolled during outbreaks between May 2014 and October 2015 in the First Affiliated Hospital, College of Medicine, Zhejiang University and Hangzhou Children's Hospital. The cases of EV71-associated HFMD were verified by clinical syndromes (such as fever, herpes of hand, foot, and/or mouth) and laboratory results, which were confirmed using the quantitative real-time RT-PCR assay. For detection of EV71 in their stools, throat swabs, vesicular fluids, or cerebrospinal fluids (CSF), the enterovirus 71 nucleic acid detection kit (Da An Gene Co., Ltd., Guangzhou, China) was used for detecting the VP1 gene of EV71. Mild cases were defined by typical clinical features and spontaneously recovered within one week. Severe cases were usually defined in accordance with previous reports [4, 26]. Clinical data and specimens were collected from the patients with EV71-associated HFMD., and Ppatients were excluded if they were also infected by bacteria before treatment, but specimens were. Forty-six healthy donors that were age and gender matched in this study were recruited as healthy controls (HC) from health checks in our hospitals. According to the Declaration of Helsinki (1964), written informed consent from the patients and HC was obtained prior to collection of clinical data and specimens. This study was approved by the Medical Ethics Committee at our hospitals. The clinical data and laboratory results of these children are shown in Table 1.

### Cell isolation

Peripheral whole blood specimens were collected from the HC and patients during the acute and convalescence stages of EV71-associated HFMD, and peripheral blood mononuclear cells (PBMCs) were isolated by density gradient centrifugation using Ficoll-Hypaque solution (CL5020, CEDARLANE, The Netherlands). Human CD4^+^T cells were isolated from PBMCs using the human CD4^+^T Cell Isolation Kit (130-096-533) (Miltenyi Biotec GmbH, Bergisch Gladbach, Germany) according to the manufacturer's protocol.

### Flow cytometric analysis

Human PBMCs were washed by phosphate buffered saline (PBS) and re-suspended in complete RPMI 1640 medium (10% fetal bovine serum, 100 U/ml penicillin and 100 μg/ml streptomycin) (Gibco, NY, USA). The cells were then stimulated for 5 h using 50 ng/ml phorbol 12-myristate 13-acetate (PMA), 1 μg/ml ionomycin and 500 ng/ml monensin (eBscience, San Diego, CA, USA) in 24-well plates. The stimulated PBMCs were stained with phycoerythrin (PE)-conjugated anti-human CD4 monoclonal antibody (mAb) at 4 °C for 30 min. These cells were fixed and permeabilized with IC fixation/permeabilization buffer (eBioscience, San Diego, CA, USA) and were intracellularly stained with allophycocyanin (APC)-conjugated anti-human IL-17A mAb and fluorescein isothiocyanate (FITC)-conjugated anti-human IL-22 mAb according to the manufacturer's protocols. Isotype-specific antibody controls were used in all procedures. All antibodies used in flow cytometric analysis were obtained from Biolegend (Biolegend, San Diego, CA, USA), and isotype-matched antibody controls were used in all procedures. Flow cytometric acquisition was performed using the FACSCalibur flow cytometer and CELLQUEST software (Becton Dickinson, Sparks, MD, USA), and data were analyzed by FlowJo software, version 7.6.5 (TreeStar, San Carlos, CA, USA).

### RNA extraction and quantitative real-time PCR

To detect the mRNA expression levels of the cytokines IL-22, IL-17A, IL-23, IL-6, and TNF-α and mRNA levels of the transcription factors AHR and RORγt, total RNA from human CD4^+^T cells and PBMCs was extracted by the QIAGEN RNeasy Mini Kit (74104) (QIAGEN GmbH, Hilden, Germany). cDNA was synthesized by a reverse transcription reagent kit (Takara, Dalian, China) according to the manufacturer's protocol. Real-time PCR was performed in triplicate using the QuantiFast™ SYBR Green PCR Kit (QIAGEN GmbH, Hilden, Germany) depending on the ABI 7500 analysis system (Applied Biosystems, Foster City, CA, USA). The thermocycle program was as follows: 5 min at 95°C for denaturation, and then 40 cycles (95°C for 10 s and 60°C for 40 s) for PCR amplification. The fluorescence values were obtained at 60°C within each cycle. The primers were as follows: IL-22: sense, 5′- GTTCCAGCCTTATATGCAGGAGG-3′, antisense, 5′-GCACATTCCTCTGGATATGCAGG-3′; IL-17A: sense, 5′-CGGACTGTGATGGTCAACCTGA-3′, antisense, 5′-GCACTTTGCCTCCCAGATCACA-3′; IL-23: sense, 5′-GAGCCTTCTCTGCTCCCTGATA-3′, antisense, 5′-GACTGAGGCTTGGAATCTGCTG-3′; IL-6: sense, 5′-AGACAGCCACTCACCTCTTCAG-3′, antisense, 5′-TTCTGCCAGTGCCTCTTTGCTG-3′; TNF-α: sense, 5′-CTCTTCTGCCTGCTGCACTTTG-3′, antisense, 5′-ATGGGCTACAGGCTTGTCACTC-3′; RORγt: sense, 5′-GAGGAAGTGACTGGCTACCAGA-3′, antisense, 5′-GCACAATCTGGTCATTCTGGCAG-3′; AHR: sense, 5′-GTCGTCTAAGGTGTCTGCTGGA-3′, antisense, 5′-CGCAAACAAAGCCAACTGAGGTG-3′. RNA expression level was normalized by the glyceraldehyde 3-phosphate dehydrogenase (GAPDH) gene described in a previous report [28]: sense, 5′-GTCTCCTCTGACTTCAACAGCG-3′; antisense, 5′-ACCACCCTGTTGCTGTAGCCAA-3′. The data were analyzed by ABI 7500 software, v2.0.5 (Applied Biosystems, Foster City, CA, USA). The experiments were performed in triplicate in this study.

### Analysis of plasma cytokines

The plasma was separated from the blood of each individual and was used to detect the levels of the cytokines IL-22, IL-17A, IL-23, IL-6, and TNF-α using the Bio-Plex Pro Human Th17 Cytokine Panel assay (Bio-Rad Laboratories, Shanghai, China) according to the manufacturer's protocols. Briefly, 50 μl of diluted (1:1) beads coated with anti-human antibodies against the corresponding cytokine antigen was mixed with 150 μl of each diluted sample (50 μl of serum; standards or controls were diluted with 100 μl of dilution buffer) and then incubated for 1 h at room temperature with vigorous shaking. After washing the plate, 25 μl of biotinylated detection antibodies was added and incubated for 30 min. After a wash cycle, 50 μl streptavidin (SA)-PE was added to the beads and incubated for 10 min. After washing the plate, the bead mixtures were re-suspended using 125 μl assay buffer and measured using the Bio-Plex 200 system (Bio-Rad Laboratories, Inc., Munich, Germany). All samples were tested in triplicate in this study. The data were analyzed using BioPlex Manager 6.1 software (Bio-Rad Laboratories, Inc., USA).

### Statistical analysis

An overall statistically significant variation among the groups was analyzed by one-way ANOVA. The difference between two groups was appropriately performed using the Student's unpaired or paired *t-*test. For non-parametric data, the Mann-Whitney U test was used for statistical change between groups. Correlations between variables were determined by Spearman's correlation coefficient. Data were analyzed with GraphPad Prism 5 software and SPSS17.0 software. *P* < 0.05 was considered statistically significant.

## Abbreviations

EV71: Enterovirus-71
HFMD: hand, foot, and mouth disease
IL: interleukin
Th: T helper cell
Th22: IL-22-producing Th cell
Th17: IL-17A-producing Th cell
cTh22: circulating Th22 cell
cTh17: circulating Th17 cell
TFH: T follicular helper
PBS: phosphate-buffered saline
PBMCs: peripheral blood mononuclear cells
TNF-α: tumor necrosis factor-α
ELISA: enzyme-linked immunosorbent assay
AHR: aryl hydrocarbon receptor
RORγt: retinoid-related orphan nuclear receptor-γt
RA: rheumatoid arthritis
SLE: systemic lupus erythematosus
MS: multiple sclerosis
IBD: inflammatory bowel disease
WBC: White blood cell
CRP: C-reactive protein
CK-MB: Creatine kinase-MB
AST: aspartate aminotransferase
ALT: alanine aminotransferase
LDH: lactate dehydrogenase
HC: healthy controls
PCR: polymerase chain reaction
